# The Influence of Leisure Attitudes and Leisure Satisfaction on Adolescents’ Positive Functioning: The Role of Emotion Regulation

**DOI:** 10.3389/fpsyg.2018.01349

**Published:** 2018-08-03

**Authors:** Teresa Freire, Ana Teixeira

**Affiliations:** School of Psychology, University of Minho, Braga, Portugal

**Keywords:** leisure attitude, leisure satisfaction, positive functioning, emotional regulation, adolescents

## Abstract

Scientific research on leisure has proven its contribution to physical and psychological well-being in adolescents, especially regarding the practice of structured leisure activities. Leisure is considered a privileged context for adolescents to develop and learn several developmental skills, such as emotion regulation (ER). Nevertheless, the relationship between leisure and ER has been under-researched in adolescents. The present cross-sectional study aims to test a conceptual model concerning the relationship of leisure attitudes and leisure satisfaction with adolescents’ positive functioning and to explore the role of emotion self-regulation strategies in that relationship. Thus, we hypothesized that leisure attitudes would be a predictor of leisure satisfaction; leisure satisfaction would be a predictor of positive functioning dimensions (self-esteem; satisfaction with life; psychological well-being); and the relationship between leisure satisfaction and positive functioning dimensions would be mediated by emotion self-regulation strategies [cognitive reappraisal (CR) and expressive suppression (ES)]. The participants in this study were 654 adolescents from 10th, 11th, and 12th grade, aged between 14 and 19 years old. Data were collected using self-report questionnaires. The structural equation analysis showed that leisure attitudes are a significant predictor of leisure satisfaction, and that leisure satisfaction significantly predicts all positive functioning dimensions. CR mediated the relationship between leisure satisfaction and self-esteem. These findings highlight the importance of developing positive attitudes toward leisure to increase adolescents’ levels of leisure satisfaction. This study also supports the importance of leisure satisfaction for achieving adolescents’ positive functioning. Future studies should continue to examine the role of emotion self-regulation strategies on leisure, especially regarding CR.

## Introduction

Leisure has been considered one of the most impactful experiences in adolescents’ lives. The benefits of leisure, understood as a context, an activity, or an experience, is well documented in the literature, along with the evidence that it (leisure) can be simultaneously as good as it is adverse (Caldwell, 2005; Caldwell and Faulk, 2013; Freire, 2017). Because of these opposing views, research has been considering the internal (individual and perceived characteristics) and external (activities, contexts, and environments) conditions that affect future life trajectories that begin in adolescence (Witt and Crompton, 2003; Freire et al., 2016). Studies on leisure activities and related lived experiences show that different consequences exist, in terms of adolescents’ growth and developmental pathways, according to the type of leisure activities they are involved in (Mahoney et al., 2005; Witt and Caldwell, 2005; Freire, 2013). According to studies supporting the role of leisure in adolescent development, it becomes more than relevant to deeply analyze the relations between variables that can, from a psychological point of view, add knowledge and evidence on the developmental processes underlying day-to-day leisure and lived experiences of adolescents. An emphasis on the leisure experiences and positive functioning of adolescents will be, therefore, the focus of the present study.

In the present study, several concepts from the perspective of leisure experience have been targeted for further study and analysis, such as leisure attitudes and leisure satisfaction. Outside the lens of leisure, and from a psychological perspective, adolescence has long been considered a pivotal moment in developmental trajectories. Recently, a new approach based on thriving and positive development has emerged, highlighting the role of developmental assets (Benson, 2007; Scales et al., 2017). Along with this perspective, researchers have tried to search for and validate the kind of factors that can increase or decrease positive life experiences, or factors that can promote potentialities and prevent risks. One such useful factor to help further understand adolescents’ thriving and development is the ecological perspective (c.f. Bronfenbrenner, 1994). Leisure and its components also appear as one of these main factors that cross developmental experiences with lived contexts, thus contributing to not only youth problem prevention but also to the thriving and growing processes that appear along the pathway to adulthood (Witt and Crompton, 2003; Freire and Stebbins, 2011).

Supported in these cutting-edge approaches, the aim of our study is twofold: (i) to know how leisure experience, expressed in terms of leisure attitudes and satisfaction, is associated with the experience of positive functioning in adolescents, understood in terms of self-esteem, life satisfaction, psychological well-being, and emotion regulation (ER); and (ii) to know how the use of ER strategies [cognitive reappraisal (CR) and cognitive suppression] mediate the relationships between leisure satisfaction and those positive functioning dimensions.

## Leisure Experience and Positive Functioning in Adolescents: Theoretical Framework and Hypothesis Formulation

Leisure experience is a complex concept in literature (see Lee et al., 1994), with several scholars and researchers trying to define it and its importance in adolescent development (Kleiber et al., 1986; Caldwell and Witt, 2011). From an ecological perspective, Freire (2013) discussed leisure experience in relation to daily subjective experience, focusing on understanding how individuals manage and conceptualize the real world, day-by-day, across their lived contexts, activities, social relations, and individual characteristics, and how these leisure experiences express and influence these processes.

The present study aims to test a conceptual model of leisure experience and positive functioning in adolescents, based on the relationships among several variables/constructs supported by empirical literature, to be presented in the following sections. For this, we analyzed two particular leisure variables that are related to leisure experience, namely, leisure attitudes and leisure satisfaction. In addition, in terms of positive functioning, we analyzed the specific variables of self-esteem, satisfaction with life, psychological well-being, and ER. Regarding ER, we highlighted the relevance of ER strategies as mediators in the relationship between leisure satisfaction and positive functioning. To the best of our knowledge, this conceptual model is the first to explore the relationships between the components of leisure experience and the components of positive functioning when mediated by ER strategies in adolescents.

### Relating Leisure Attitudes and Leisure Satisfaction in Adolescents

In the field of leisure studies, leisure attitudes and leisure satisfaction have emerged as central issues for their contribution to a better understanding of leisure experience and involvement or participation in leisure activities.

The concept of leisure attitude was primarily developed by Ragheb and Beard (1982). The authors tried to understand individuals’ willingness or predisposition to engage in leisure activities, which they found to be influenced by the individuals’ attitudes to leisure, according to its cognitive, affective, and behavioral components. Those same authors developed the concept of leisure satisfaction (Beard and Ragheb, 1980), which they defined as the positive perceptions or feelings individuals form (elicit or gain) as a result of engaging in leisure activities. In their own words, “[Leisure satisfaction] is the degree to which one is presently content or pleased with his/her general leisure experiences and situations” (p. 22). For the authors, leisure satisfaction is related to six dimensions, including psychological, educational, social, relaxational, physiological, and aesthetic dimensions, which then becomes the measure of the extent to which individuals perceive that certain personal needs are met or satisfied through leisure activities (Beard and Ragheb, 1980). Although some studies consider the different dimensions as either causes or effects with other variables, the present study will consider the whole concept as a result of the six dimensions taken together as a measure of leisure satisfaction.

The relationship between leisure attitude and leisure satisfaction has already been documented by Ragheb and Tate (1993). In the same line, other studies found a positive association between leisure attitudes and leisure satisfaction with college student samples (Kim et al., 2015; Choi and Yoo, 2017), showing that studies with adolescents are still scarce (Freire and Fonte, 2007; Teixeira and Freire, 2013).

Our conceptual model departed from the relationship between leisure attitude and leisure satisfaction, assuming that leisure attitudes would be significantly associated with leisure satisfaction. A positive leisure attitude tends to improve serious and motivated involvement in activities, generating leisure satisfaction (Ragheb and Tate, 1993; Kim et al., 2015; Choi and Yoo, 2017). Therefore, we expected that a favorable attitude to leisure would lead to a higher level of leisure satisfaction in adolescents.

Hypothesis 1: Leisure attitude has a direct effect on leisure satisfaction in adolescents.

### From Leisure Satisfaction to Positive Functioning, and the Role of the Emotion Regulation Strategies in Adolescents

Several studies have shown how leisure satisfaction is associated with other individual life dimensions, underlying the importance of these associations in adolescent development. According to Kim et al. (2015), the satisfaction obtained from the participation in leisure activities, and also having positive attitudes toward leisure, have been associated with increases in self-esteem. Other studies have found a direct effect of leisure satisfaction on self-esteem in undergraduate student and older adult samples (Tsai et al., 2012; Kim et al., 2015). Also, recent research has focused on the benefits of leisure attitudes and leisure satisfaction for adolescents’ levels of well-being and overall life satisfaction (Haworth and Lewis, 2005; Freire and Fonte, 2007; Wang et al., 2008). According to Shin and You (2013), leisure satisfaction has a longitudinal effect on psychological well-being and life satisfaction for adolescents. For Choi and Yoo (2017), leisure satisfaction is also related to life satisfaction. Trainor et al. (2010) studied the link between adolescents’ leisure participation and psychological well-being – understood as higher scores on measures of self-esteem and life satisfaction, positive mood states, and the absence of significant levels of depression, anxiety, and stress. In their study, dispositional variables were better predictors of adolescent well-being than spare-time use was. Also, the types of activities adolescents become involved in emerged as an important issue in understanding psychological well-being. According to all these studies, it seems clear that leisure, and specifically leisure satisfaction, can have an impactful role in enhancing positive functioning, expressed in terms of self-esteem, life satisfaction, and psychological well-being, with evident relationships between them. The latter three variables are those that are under investigation in our study.

Fewer studies tend to be found on the relationships between leisure experience and emotions, and more specifically, on ER. A widely accepted definition of ER was presented by Thompson (1994), stating that “emotion regulation consists of the extrinsic and intrinsic processes responsible for monitoring, evaluating, and modifying emotional reactions, especially their intensive and temporal features, to accomplish one’s goals” (pp. 27–28). According to Zeman et al. (2006), researchers are shifting their focus from emphasizing the investigation of discrete emotion states to examining the features of ER and the role of emotions in producing constructive, adaptive behaviors and outcomes, along with development. For this, ER has been considered one of the most robust and critical constructs in child development and a central topic in the positive developmental perspective of adolescents (Zeman et al., 2006; Riediger and Klipker, 2014; Morrish et al., 2017), though there are few to no studies directly relating it with leisure.

Morrish et al. (2017) highlighted the importance of ER across adolescent development, as it promotes psychological flexibility, resilience, and well-being in youth. Considering positive functioning and the evidence based on findings of positive development, they also underlined the role of ER in incrementing positive affect, which in turn plays an important role in maintaining and improving psychological well-being. Understanding causes and consequences of ER in adolescents implies targeting its relationship with contexts and situations where emotions will emerge. However, emotions can be regulated in several different ways, and being adaptive or maladaptive depends on the specific social demands of the situation (Zeman et al., 2006). Following this perspective, leisure activities and related experiences make leisure a relevant context from which to develop, socialize, and practice ER strategies. Due to the positive impact leisure can have on adolescent development, we must consider its value for the healthy development of ER processes and the use of ER strategies. However, no studies could be found studying ER associated with leisure, or leisure experience in adolescents.

The model of ER from Gross (1998, 2014) has been widely used in the study of ER in adolescents. Although his model can distinguish between different strategies, empirical literature has shown the impact of two main strategies in adolescent psychosocial functioning: the CR and the expressive suppression (ES). These two strategies have been receiving great empirical attention. CR implies changing the way we think about an emotion-eliciting situation to decrease its emotional impact, while ES refers to an effort to hide or inhibit the emotion expressive behavior (Tavares and Freire, 2016; Morrish et al., 2017). A recent study on psychosocial well-being in adolescence was focused on analyzing whether or not these ER strategies were associated with adolescents’ well-being (Verzeletti et al., 2016). ER strategies are related to several well-being indicators, such as affect, loneliness, psychological health, and life satisfaction, showing different patterns of associations between those indicators and each one of the strategies. The adolescents’ greater use of CR was associated with higher levels of psychosocial well-being, and their use of ES was associated with negative outcomes in well-being. In a similar vein, Teixeira et al. (2015) found that CR was positively associated with self-esteem and satisfaction with life, and in turn, ES was negatively associated with those same variables.

To know how these ER strategies are used, practiced, or chosen needs further research, and looking to leisure could be the real-life laboratory needed. Therefore, and besides studying how ER strategies can be associated with each one of the positive functioning variables, we were also interested in their mediating role between leisure satisfaction and positive functioning variables. Due to the role of ER strategies in the way individuals regulate their emotions in connection with the contexts for achieving their desired goals, we expected that these two strategies could mediate the relationships of leisure satisfaction and the three positive functioning variables: self-esteem, life satisfaction, and psychological well-being. Thus, in line with the quoted literature findings, and according to our perspective on positive development and optimal functioning in adolescence, we hypothesized the following:

Hypothesis 2: Leisure satisfaction has a direct effect on self-esteem, satisfaction with life, and psychological well-being.
Hypothesis 3: Leisure satisfaction has a direct effect on the use of ER strategies (CR and ES).
Hypothesis 4: Each one of the ER strategies (CR and ES) has a direct effect on each one of the positive functioning variables (self-esteem, life satisfaction, and psychological well-being).
Hypothesis 5: Each of the ER strategies (CR and ES) mediates the relationship of leisure satisfaction with self-esteem, satisfaction with life, and psychological well-being.

**FIGURE 1:**
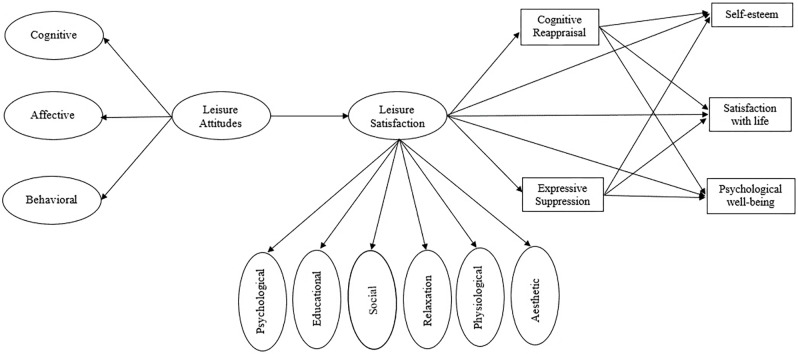
The proposed conceptual model.

The answers to these hypotheses, represented in our conceptual model (**Figure 1**), will contribute to the knowledge on how leisure experiential outcomes (such as leisure attitudes and satisfaction) can be associated with self-esteem, satisfaction with life, and psychological well-being (understood as dimensions of adolescents’ positive functioning), and how the use of ER strategies (CR and ES) mediate those relationships. Due to the lack of evidence on some of these relationships, this paper describes an explorative study. Thus, the way we conceived the various concepts underlying these variables (as discussed in previous sections) modelled a unidirectional way of relationships to be tested through the presented model. Although other models of relationships will be possible, we aimed for the contribution to new insights on positive functioning in adolescence as a consequence of one of the most significant life experiences in adolescence: leisure.

## Materials and Methods

### Participants

A total of 654 Portuguese adolescents from secondary school participated in the current study. Adolescents’ ages ranged from 14 to 19 years old (*M* = 16.25, *SD* = 1.06). Age frequency was according to the following: 12 participants were 14 years old (1.8%); 171 were 15 years old (26.1%); 195 were 16 years old (29.8%); 203 were 17 years old (31%); 62 were 18 years old (9.5%); and 11 were 19 years old (1.7%). The sample was balanced according to the school year and gender of the participants. **Table 1** presents the demographic characteristics of the sample. Moreover, 338 participants (51.7%) were engaged in structured leisure activities (such as sports, artistic, religious, social, or volunteering activities), and 316 (48.3%) were engaged in non-structured leisure activities (such as watching TV, talking, reading, or playing videogames).

**Table 1:** Demographic characteristics.

School grade	Gender			
	Boys *N* (%)	Girls *N* (%)	Total *N* (%)	Age (years) *M* (*SD*)
10th year	109 (16.67%)	109 (16.67%)	218 (33.33%)	15.21 (0.57)
11th year	109 (16.67%)	109 (16.67%)	218 (33.33%)	16.25 (0.69)
12th year	109 (16.67%)	109 (16.67%)	218 (33.33%)	17.30 (0.59)
Total	327 (50%)	327 (50%)	654 (100%)	16.25 (1.06)


### Procedures

The present study has a cross-sectional design. The data were collected at a Portuguese high school from an urban context, using convenience sampling. Researchers presented the study to the school staff and students who gave their informed consent. The school staff informed the parents of the participating students and collected their informed consent. The questionnaires were administered to the students during class time, by graduate students of psychology, and followed all ethical and deontological research principles (American Psychological Association, 2010). The surveys were conducted using a counterbalanced order to avoid carryover and order effects (Foley, 2004).

### Instruments

#### Sociodemographic Questionnaire

The participants answered a brief survey to assess participants’ age, gender, school year, nationality, and the leisure activities they engaged in.

#### Leisure Attitudes

The Leisure Attitude Scale – Short Version (LAS-SV, Ragheb and Beard, 1982; Portuguese validation of Teixeira and Freire, 2013) is an 18-item self-report measure that assesses attitudes toward leisure. It includes three components of leisure attitude with six items in each: cognitive (item example: “Engaging in Leisure activities is a wise use of time”), affective (item example: “Leisure activities give me pleasure”), and behavioral (item example: “I spend considerable time and effort to be more competent in my leisure activities”). The items are answered using a 5-point Likert-type response scale (from 1 = strongly disagree to 5 = strongly agree). The total score of the LAS-SV ranges from 18 to 90 and the total score of each attitude component ranges from 6 to 30. Higher scores indicate positive attitudes toward leisure. In this study, reliability analysis showed a Cronbach’s alpha of 0.90 for the total scale and an alpha of 0.87, 0.89, and 0.78 for the cognitive, affective, and behavioral subscales, respectively.

#### Leisure Satisfaction

The Leisure Satisfaction Scale (LSS, Beard and Ragheb, 1980; Portuguese validation of Teixeira, 2013) is a 24-item self-report scale that assesses the leisure satisfaction levels of individuals. This scale includes six subscales with four items in each: psychological (item example: “My leisure activities are interesting to me”); educational (item example: “My leisure activities increase my knowledge about things around me”); social (item example: “I have social interaction with others through leisure activities”); relaxation (item example: “My leisure activities help me to relax”); physiological (item example: “My leisure activities are physically challenging”); and aesthetical (item example: “The areas or places where I engage in my leisure activities are fresh and clean”). The items are answered based on a 5-point Likert-type scale (from 1 = almost never true to 5 = almost always true). The total score ranges from 24 to 96, and the subscales scores range from 4 to 16. Higher scores indicate higher levels of leisure satisfaction. In this study, the Cronbach’s alpha results were as follows: total scale = 0.93; psychological = 0.84; educational = 0.81; social = 0.81; relaxation = 0.83; physiological = 0.91; and aesthetical = 0.84.

#### Self-Esteem

The Rosenberg Self-Esteem Scale (RSES; Rosenberg, 1965; Portuguese adolescent validation of Romano et al., 2007) is a 10-item self-report questionnaire that assesses general feelings of self-esteem (item example: “On the whole, I am satisfied with myself”). The items were answered using a 4-point Likert-type response scale (from 1 = strongly disagree to 4 = strongly agree). Total scores can range between 10 and 40 points. Higher scores indicate higher levels of self-esteem. In this study, this scale presented a Cronbach’s alpha of 0.87.

#### Satisfaction With Life

The Satisfaction with Life Scale (SWLS; Diener et al., 1985; Portuguese adolescent validation of Neto, 1993) assesses global cognitive judgments of satisfaction with life (item example: “In most ways, my life is close to my ideal”). The five items of this scale are rated using a 7-point Likert-type response scale (from 1 = strongly disagree to 7 = strongly agree). Total scores range between 5 and 35 points. Higher scores indicate higher levels of satisfaction with life. In the present study, this scale presented a Cronbach’s alpha of 0.85.

#### Psychological Well-Being

The Psychological Well-Being Scale for Adolescents (EBEPA, Bizarro, 1999) is a 28-item self-report scale explicitly developed for the assessment of psychological well-being in adolescents (item example: “I thought I was able to do things as well as others”). The items are answered using a 6-point Likert-type response scale regarding the self-evaluation of the frequency of occurrence (from 1 = “never” to 6 = “always”). This instrument has five subscales and an index of total well-being. In this study, only the total scores of well-being were used, which presented a Cronbach’s alpha of 0.88.

#### Emotion Regulation Strategies

The Emotion Regulation Questionnaire – Children and Adolescents (ERQ-CA, Gullone and Taffe, 2012; Portuguese validation of Teixeira et al., 2015) assess the use of two ER strategies: CR and ES. The 10 items of this scale are answered using a 5-point Likert scale (from 1 = strongly disagree to 5 = strongly agree). The CR subscale included six items (item example: “When I want to feel happier, I think about something different”) and the ES subscale includes four items (item example: “I keep my feelings to myself”). Total scores are calculated by the sum of all items, ranging from 6 to 30 in the CR subscale and from 4 to 20 in the ES subscale. Higher scores indicate a greater use of the strategy. In this study, reliability analysis presented a Cronbach’s alpha of 0.76 for the CR and 0.65 for the ES.

### Data Analysis

First, SPSS software (v. 22.0) was used to generate descriptive statistics (means and standard deviations) and reliability analyses for all scales. Then, Rstudio software (v. 0.97) and Lavaan package (Rosseel et al., 2013) were used to perform confirmatory factor analyses (CFA) to test the factor structure of all the scales resorting to modification indices; and to conduct a structural equation model (SEM) to examine the direct and indirect relationships suggested by the proposed model. The analyses were performed using maximum likelihood estimates. Missing values were handled using the full information maximum likelihood (FIML) method. Model fit was assessed by the following criteria: ratio chi-square statistics/degrees of freedom (*X*^2^/*df*); goodness-of-fit index (GFI), which measures the relative amount of variance and covariance jointly explained by the model; adjusted goodness-of-fit index (AGFI) that assesses parsimony of the model; comparative fit index (CFI) that evaluates the adequacy of the model relative to the independence model; and root means square error of approximation (RMSEA), which evaluates the discrepancy between the estimated and the obtained matrices (Byrne, 2001). Regarding the first index, Byrne (1989) suggests that values between 2.00 and 5.00 define appropriate adjustment. Values over 0.90 indicate an adequate adjustment for GFI, AGFI, and CFI (Bentler and Bonnet, 1980; Byrne, 2001). Values lower than 0.08 are indicative of an acceptable fit for RMSEA (Schreiber et al., 2006). The Bayesian Information Criterion (BIC) was used to compare models, where the model with the lowest value is considered the preferable model.

## Results

### Descriptive Statistics

According to leisure dimensions (leisure satisfaction and attitudes), on average, participants revealed high levels of satisfaction and positive attitudes toward leisure. The mean scores for positive functioning dimensions (self-esteem, satisfaction with life, and psychological well-being) also showed medium to high scores. As in other studies (Gullone and Taffe, 2012; Teixeira et al., 2015), participants scored higher in CR than ES, revealing a higher use of the first strategy when compared to the second. **Table 2** shows all these values.

**Table 2:** Descriptive statistics and reliability values for the scales.

Scales and subscales	No. of items	Mean	SD	Min.–Max.
Leisure Attitude (total)	18	4.06	0.53	1–5
Cognitive	6	4.33	0.60	1–5
Affective	6	4.28	0.63	1–5
Behavioral	6	3.57	0.72	1–5
Leisure Satisfaction (total)	24	3.94	0.57	1.33–5
Psychological	4	4.05	0.71	1–5
Educational	4	3.95	0.71	1–5
Social	4	3.85	0.76	1–5
Relaxation	4	4.19	0.69	1–5
Physiological	4	3.67	0.99	1–5
Aesthetic	4	3.89	0.74	1–5
Self-esteem (total)	10	3.09	0.52	1.10–4
Satisfaction with life (total)	5	4.57	1.36	1–7
Psychological well-being (total)	28	4.40	0.64	1.79–5.71
Cognitive reappraisal (total)	6	3.59	0.64	1.17–5
Expressive suppression (total)	4	2.99	0.74	1–5


### Correlational Analysis

**Table 3** shows Pearson correlations among the variables in this study. In general, the Leisure scales and subscales also correlated positively with positive dimensions. Only the behavioral attitude (planned actions to leisure activities or experiences) was not associated with life satisfaction. Results also showed significant correlations between leisure scales and related subscales with CR. However, no associations were found between those leisure scales and related subscales with ES. CR was positively correlated with all positive dimensions except with life satisfaction. On the other hand, ES presented significant negative correlations with all positive dimensions and a positive correlation with CR.

**Table 3:** Correlation between Leisure Satisfaction scale and subscales and other scales.

	1	2	3	4	5	6	7	8	9	10	11	12	13	14	15	16
(1) Leisure Satisfaction	–	0.84^∗∗∗^	0.81^∗∗∗^	0.72^∗∗∗^	0.71^∗∗∗^	0.73^∗∗∗^	0.69^∗∗∗^	0.63^∗∗∗^	0.51^∗∗∗^	0.57^∗∗∗^	0.48^∗∗∗^	0.19^∗∗∗^	0.20^∗∗∗^	0.29^∗∗∗^	0.17^∗∗∗^	0.01
(2) Psychological		–	0.66^∗∗∗^	0.54^∗∗∗^	0.60^∗∗∗^	0.52^∗∗∗^	0.49^∗∗∗^	0.60^∗∗∗^	0.45^∗∗∗^	0.57^∗∗∗^	0.45^∗∗∗^	0.17^∗∗∗^	0.20^∗∗∗^	0.26^∗∗∗^	0.15^∗∗∗^	-0.01
(3) Educational			–	0.55^∗∗∗^	0.51^∗∗∗^	0.47^∗∗∗^	0.48^∗∗∗^	0.51^∗∗∗^	0.40^∗∗∗^	0.44^∗∗∗^	0.41^∗∗∗^	0.14^∗∗^	0.16^∗∗∗^	0.20^∗∗∗^	0.13^∗∗^	-0.02
(4) Social				–	0.40^∗∗∗^	0.39^∗∗∗^	0.38^∗∗∗^	0.44^∗∗∗^	0.34^∗∗∗^	0.35^∗∗∗^	0.39^∗∗∗^	0.16^∗∗∗^	0.16^∗∗∗^	0.21^∗∗∗^	0.09^∗^	-0.01
(5) Relaxation					–	0.35^∗∗∗^	0.43^∗∗∗^	0.52^∗∗∗^	0.50^∗∗∗^	0.53^∗∗∗^	0.28^∗∗∗^	0.11^∗∗^	0.13^∗∗^	0.19^∗∗∗^	0.12^∗∗^	-0.03
(6) Physiological						–	0.36^∗∗∗^	0.38^∗∗∗^	0.30^∗∗∗^	0.32^∗∗∗^	0.31^∗∗∗^	0.17^∗∗∗^	0.13^∗∗^	0.22^∗∗∗^	0.13^∗∗^	-0.04
(7) Aesthetic							–	0.42^∗∗∗^	0.32^∗∗∗^	0.40^∗∗∗^	0.32^∗∗∗^	0.12^∗∗^	0.15^∗∗∗^	0.23^∗∗∗^	0.17^∗∗∗^	0.06
(8) Leisure Attitude								–	0.82^∗∗∗^	0.85^∗∗∗^	0.79^∗∗∗^	0.16^∗∗∗^	0.12^∗∗^	0.25^∗∗∗^	0.16^∗∗∗^	0.02
(9) Cognitive									–	0.66^∗∗∗^	0.41^∗∗∗^	0.15^∗∗∗^	0.14^∗∗∗^	0.27^∗∗∗^	0.15^∗∗∗^	0.00
(10) Affective										–	0.46^∗∗∗^	0.14^∗∗∗^	0.15^∗∗∗^	0.23^∗∗∗^	0.12^∗∗^	0.02
(11) Behavioral											–	0.09^∗^	0.01	0.13^∗∗^	0.12^∗∗^	0.04
(12) Self-esteem												–	0.29^∗∗∗^	0.55^∗∗∗^	0.15^∗∗∗^	-0.10^∗^
(13) Satisfaction with life													–	0.42^∗∗∗^	0.05	-0.09^∗^
(14) Psychological well-being														–	0.10^∗^	-0.12^∗∗^
(15) Cognitive reappraisal															–	0.14^∗∗^
(16) Expressive suppression																–


*^∗^*p* < 0.05; ^∗∗^*p* < 0.01; ^∗∗∗^*p* < 0.001.*

### The Measurement Model

First, we performed a two-order CFA to test the factorial structure of the Leisure Attitudes Scale and the LSS. The Leisure Attitudes Scale presented a good model fit (*X*^2^ = 392.001, *p* = 0.001; *df* = 132; *X*^2^/*df* = 2.970; CFI = 0.949; RMSEA = 0.056; GFI = 0.991; AGFI = 0.988). As well, CFA results of the LSS showed a good model fit (*X*^2^ = 841.912, *p* = 0.001; *df* = 246; *X*^2^/*df* = 3.422; CFI = 0.926; RMSEA = 0.064; GFI = 0.981; AGFI = 0.975). Then, we tested a measurement model assuming correlation between both scales. Results showed a good model fit (*X*^2^ = 1960.183, *p* = 0.001; *df* = 809; *X*^2^/*df* = 2.423; CFI = 0.911; RMSEA = 0.050; GFI = 0.969; AGFI = 0.963). Both scales presented a correlation coefficient of 0.774. **Table 4** shows the constructs and respective indicators of both scales and the standardized factor loadings of the CFA’s and measurement model.

**Table 4:** Confirmatory factor analysis and measurement model.

First-order construct	Second-order construct	Indicators	CFA	Measurement model
			Standardized loadings	Standardized loadings
Leisure Attitude	Cognitive		0.822	0.802
		Item 1	0.718	0.696
		Item 2	0.734	0.746
		Item 3	0.724	0.732
		Item 4	0.751	0.738
		Item 5	0.719	0.743
		Item 6	0.730	0.727
	Affective		0.900	0.881
		Item 7	0.773	0.760
		Item 8	0.835	0.825
		Item 9	0.788	0.767
		Item 10	0.678	0.664
		Item 11	0.684	0.686
		Item 12	0.779	0.770
	Behavioral		0.608	0.639
		Item 13	0.636	0.633
		Item 14	0.532	0.501
		Item 15	0.601	0.617
		Item 16	0.650	0.646
		Item 17	0.656	0.637
		Item 18	0.602	0.600
Leisure Satisfaction	Psychological		0.904	0.920
		Item 1	0.715	0.726
		Item 2	0.796	0.795
		Item 3	0.848	0.848
		Item 4	0.695	0.689
	Educational		0.879	0.857
		Item 5	0.711	0.709
		Item 6	0.732	0.735
		Item 7	0.745	0.745
		Item 8	0.695	0.693
	Social		0.709	0.694
		Item 9	0.701	0.704
		Item 10	0.765	0.764
		Item 11	0.741	0.746
		Item 12	0.722	0.722
	Relaxation		0.687	0.712
		Item 13	0.829	0.831
		Item 14	0.855	0.853
		Item 15	0.802	0.801
		Item 16	0.508	0.510
	Physiological		0.620	0.610
		Item 17	0.820	0.820
		Item 18	0.925	0.928
		Item 19	0.842	0.851
		Item 20	0.838	0.840
	Aesthetic		0.647	0.653
		Item 21	0.676	0.666
		Item 22	0.807	0.803
		Item 23	0.828	0.824
		Item 24	0.742	0.736


### The Structural Equation Models

**FIGURE 2:**
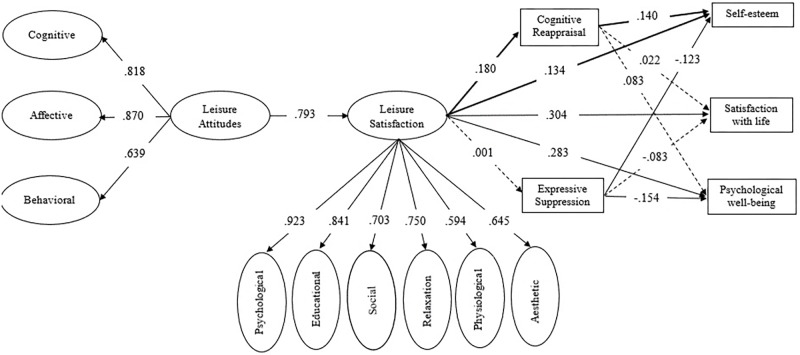
Structural equation model. The coefficients presented in the figure are standardized. Solid lines correspond to statistically significant paths (*p* < 0.05) and dotted lines corresponds to non-significant paths.

Regarding the structural model (**Figure 2**), results showed that the goodness of fit was good (*X*^2^ = 2023.197, *p* < 0.001; *df* = 1015; *X*^2^/*df* = 1.993; CFI = 0.902; RMSEA = 0.048; GFI = 0.974; AGFI = 0.970) and most of the direct and indirect paths were statistically significant (*p* < 0.05), confirming H1 and H2. H3 was only partially confirmed because leisure satisfaction had a direct effect on CR, but not on ES. As well, H4 was not totally confirmed since CR only had a direct effect on self-esteem, and ES only had a direct effect on self-esteem and psychological well-being. Regarding H5, leisure satisfaction only had an indirect effect on self-esteem through CR. The Sobel test showed that CR mediated the relationship between leisure satisfaction and self-esteem (*z* = 2.27, *p* = 0.023).

**FIGURE 3:**
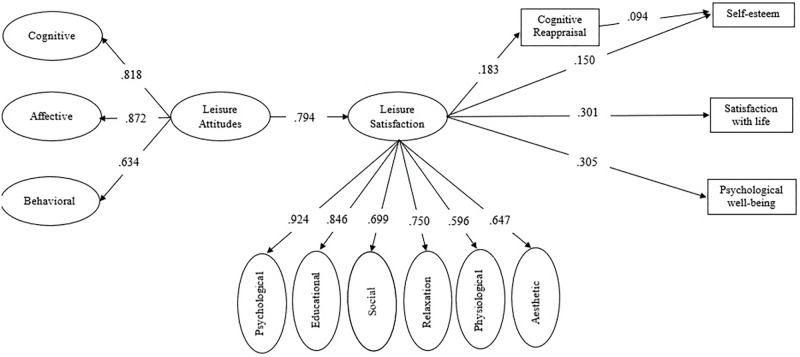
The final model. The coefficients presented in the figure are standardized. All paths were statistically significant (*p* < 0.05).

Taking into consideration the results of the SEM for the total sample, we decided to test a second model without the ES variable and the other non-significant paths. The goodness of fit of this second model were as follows: *X*^2^ = 1980.237, *p* < 0.001; *df* = 975; *X*^2^/*df* = 2.031; CFI = 0.903; RMSEA = 0.049; GFI = 0.972; AGFI = 0.967. This goodness of fit result was very similar to the first model tested, maintaining a good model fit. **Figure 3** shows the resulting model with estimated path coefficients for the total sample. In this second model, CR maintains its mediator role between leisure satisfaction and self-esteem (*z* = 1.98, *p* = 0.047).

## Discussion

The present study aimed to present and test a conceptual model of the relationships between leisure experience (attitude and satisfaction), positive functioning dimensions, and ER strategies in an adolescent sample. The main innovation of this study was to analyze the relationship between leisure satisfaction and the use of ER strategies, and how these strategies mediate the relationship between leisure satisfaction and positive functioning dimensions (understood as self-esteem, psychological well-being, and satisfaction in life). Findings supported this conceptual model showing a good model fit to the sample studied.

As expected, results have confirmed the association between leisure attitude and leisure satisfaction, showing the importance of having positive leisure attitudes in order to achieve higher levels of leisure satisfaction. This overall result is consistent with revised literature, since the primary studies of Beard and Ragheb (1980). The results, although not new, can reinforce this positive association of leisure attitudes on leisure satisfaction and, specifically, in adolescents. Most of the studies tend to use adults or young adults (e.g., college students), and so our results enlarge this pattern of positive relationship by including a younger population. Concerning leisure satisfaction itself, and as expected, our findings showed its positive contribution for higher levels of self-esteem, satisfaction with life, and psychological well-being. It is noteworthy that the association of leisure satisfaction with satisfaction with life and psychological well-being is higher as compared with the relationship with self-esteem. Overall, these results underline the important association between such a kind of leisure experience (satisfaction) and some dimensions of positive functioning, for which there is little evidence in literature. In particular, the strong association between satisfaction with life and psychological well-being can be a main contribution to the study of well-being in its multiple facets. According to literature, satisfaction with life tends to be associated with subjective well-being (Diener et al., 2012). Therefore, we could suggest our results evidence the association of leisure satisfaction on both psychological well-being and subjective well-being (Freire et al., 2013) in adolescents. The relations between subjective and psychological well-being and their interrelations with leisure experiences become a new topic to deeply explore in adolescence (Kuykendall et al., 2015). This relation between leisure satisfaction and dimensions of positive functioning is of scientific relevance, both for the field leisure as well as for positive psychology approaches to adolescent development (Freire, 2017).

In turn, positive psychological dimensions were also associated with ER strategies, showing, as expected, the relevance of studying ER in adolescents when considering their positive experiences in leisure. Our study showed, in line with literature, that the use of CR seems to be positively associated with positive variables, and ES more negatively associated with those same variables (Teixeira et al., 2015; Verzeletti et al., 2016). However, some slight differences were found: CR was only positively associated with self-esteem, and ES was negatively associated with psychological well-being and self-esteem. None of the ER strategies were directly associated with satisfaction with life, which is contrary to some literature where the CR strategy appeared positively associated to self-esteem and satisfaction with life, and the ES strategy negatively associated with those same variables (Teixeira et al., 2015). The fact that our study analyzes these associations in relation to leisure may be the core aspect to consider. To understand how leisure experiences can influence emotional regulatory mechanisms seems to be a core question that emerges from our study. These findings contribute to showing the importance of considering ER strategies when trying to understand adolescents’ emotional functioning and, at the same time, the differentiating effect it can have in regulating adolescents’ positive functioning (Zeman et al., 2006). All these findings highlight the relevance in studying how the use of these ER strategies can mediate the association between leisure satisfaction and positive functioning, putting leisure experience in the center of ER studies in adolescence. Looking at the relationship between leisure satisfaction and ER strategies, results showed that leisure satisfaction has a positive association with the use of CR, but not with ES, showing the prevalence of a cognitive strategy in detriment of a suppressive strategy when leisure experience is considered. This invalidates our expectations that leisure satisfaction would be associated with the use of both strategies. In fact, in our study, having higher levels of satisfaction in leisure promotes a higher use of the CR strategy, which in turn improves the relationship between leisure satisfaction and self-esteem, underlying the mediating role of this strategy. This is in line with new perspectives on understanding the role of emotions and their regulation in producing constructive and adaptive behaviors and outcomes, and not only their disorganization and destructive qualities (Zeman et al., 2006). Empirical studies tend to show that CR strategies have, in general, healthier effects on affective, cognitive, social functioning, and well-being than ES strategies (Butler et al., 2003; Tavares and Freire, 2016; Morrish et al., 2017). To know the how and the why leisure interferes in ER patterns, and in what direction it regulates emotional processes in adolescence, is a new research milestone. If emotions and ER processes are dynamic and highly context-dependent (Troy et al., 2013; Silva et al., 2018), leisure as an experiential context can bring new insights to ER strategies, that in turn can improve the knowledge of positive developmental processes in adolescence.

Despite the new findings, this study has some limitations that underlines the need for further research. Being a cross-sectional study, it does not allow for testing causal relationships between the variables under study. Future research should try using longitudinal designs, with a broader sample in terms of number of participants and age range. A more significant amount of data will increase the possibility to (re)test the present model and also to analyze new possible relationships between the variables under study.

Furthermore, this study has only included variables on positive leisure experiences and positive functioning dimensions. However, the relevance of the obtained results and their contribution to the topic is only one part of the picture. Future studies should also examine the relationships between leisure experiences and some other variables related to negative or distressful functioning and still try to understand the mediation role of the ER strategies. Furthermore, some empirical studies have highlighted the relevance of other variables of positive functioning that future research on leisure should also address to enhance knowledge of these processes and relationships. For instance, the type of leisure activities, the contextual or environmental features associated with leisure experiences, the socialization process of adolescents, and other related concepts about leisure (cf. Freire and Caldwell, 2013) should be studied in future research to understand if different patterns of relationships among leisure experiences, ER strategies, and positive functioning emerge. For instance, gender differences should also be considered in future research in order to see if these results are a general pattern for adolescents, or on the contrary are gender-based, thus emerging different kinds of associations between the variables for males and females.

## Conclusion

The present study has drawn attention to the study of ER strategies in the scope of leisure and positive functioning in adolescence. It suggests that positive leisure attitudes are associated with higher levels of leisure satisfaction that, in turn, are positively associated with self-esteem, satisfaction with life, and psychological well-being. Moreover, the association between leisure satisfaction and self-esteem tend to be higher when adolescents make use of the CR strategy in terms of ER. These findings shed light on new approaches for educators or professionals that work with adolescents. For example, the need to consider the effects of positive leisure experiences (attitudes and satisfaction) on improving adolescents’ positive functioning. From here, leisure education should be a main goal in the socialization process during adolescence, and in multiple life contexts (e.g., school, family, and community). According to our results, and if positive leisure experiences improve positive functioning, one can think about its role in maximizing youth potentialities through leisure. Furthermore, the use of ER processes should be considered throughout leisure experiences once they can mediate the association between leisure experience and positive functioning. Concretely, our study highlighted the association between cognitive strategy and self-esteem, highlighting a cognitive focus in ER in relation to leisure.

## Author Contributions

TF was responsible for conception and design of the study, and interpretation of data and conclusions. AT was responsible for analysis and interpretation of data. Both authors participated in drafting the article and revising it critically. Both authors give final approval of the version to be submitted.

## Conflict of Interest Statement

The authors declare that the research was conducted in the absence of any commercial or financial relationships that could be construed as a potential conflict of interest.
